# Computational simulation and target prediction studies of solubility optimization of decitabine through supercritical solvent

**DOI:** 10.1038/s41598-022-21233-0

**Published:** 2022-11-07

**Authors:** Saad M. Alshahrani, Bjad K. Almutairy, Munerah M. Alfadhel, Amany Belal, Mohammed A. S. Abourehab, Ahmed Al. Saqr, Abdullah S. Alshetaili, Kumar Venkatesan, Amal M. Alsubaiyel, Mahboubeh Pishnamazi

**Affiliations:** 1 Department of Pharmaceutics, College of Pharmacy, Prince Sattam Bin Abdulaziz University, P.O. Box 173, Al-Kharj, 11942 Saudi Arabia; 2 Department of Pharmaceutical Chemistry, College of Pharmacy, Taif University, Taif, 21944 Saudi Arabia; 3 Department of Pharmaceutics, College of Pharmacy, Umm Al-Qura University, Makkah, 21955 Saudi Arabia; 4 Department of Pharmaceutics and Industrial Pharmacy, Faculty of Pharmacy, Minia University, Minia, 61519 Egypt; 5 Department of Pharmaceutical Chemistry, College of Pharmacy, King Khalid University, Abha, 62529 Kingdom of Saudi Arabia; 6 Department of Pharmaceutics, College of Pharmacy, Qassim University, Buraidah, 52571 Saudi Arabia; 7 Institute of Research and Development, Duy Tan University, Da Nang, 550000 Vietnam; 8 The Faculty of Pharmacy, Duy Tan University, Da Nang, 550000 Vietnam; 9 Medicinal Chemistry Department, Faculty of Pharmacy, Beni-Suef University, Beni-Suef, 62514 Egypt

**Keywords:** Drug delivery, Green chemistry

## Abstract

Computational analysis of drug solubility was carried out using machine learning approach. The solubility of Decitabine as model drug in supercritical CO_2_ was studied as function of pressure and temperature to assess the feasibility of that for production of nanomedicine to enhance the solubility. The data was collected for solubility optimization of Decitabine at the temperature 308–338 K, and pressure 120–400 bar used as the inputs to the machine learning models. A dataset of 32 data points and two inputs (P and T) have been applied to optimize the solubility. The only output is Y = solubility, which is Decitabine mole fraction solubility in the solvent. The developed models are three models including Kernel Ridge Regression (KRR), Decision tree Regression (DTR), and Gaussian process (GPR), which are used for the first time as a novel model. These models are optimized using their hyper-parameters tuning and then assessed using standard metrics, which shows R^2^-score, KRR, DTR, and GPR equal to 0.806, 0.891, and 0.998. Also, the MAE metric shows 1.08E−04, 7.40E−05, and 9.73E−06 error rates in the same order. The other metric is MAPE, in which the KRR error rate is 4.64E−01, DTR shows an error rate equal to 1.63E−01, and GPR as the best mode illustrates 5.06E−02. Finally, analysis using the best model (GPR) reveals that increasing both inputs results in an increase in the solubility of Decitabine. The optimal values are (P = 400, T = 3.38E + 02, Y = 1.07E−03).

## Introduction

Production of nanomedicine has been a topic of first-rate hobby in pharmaceutical vicinity due to their importance significance for improving the drug efficacy. Nanosized drug production is one of the strategies to decorate drug solubility due to the expanded surface area to nanosized which therefore outcomes in an enhancement in drug solubility^1–3^. Given that, maximum newly discovered drugs are poorly soluble in aqueous media, underpinning studies is required to secorate the solubility of drugs thru one of the kind strategies such as nanonization, amorphous solid dispersion, crystallization, and salt formation^4–8^.

Drug nanonization has been used as an appealing technique for optimization of drugs thru solubility enhancement withinside the body. Supercritical solvents are extra appealing techniques due to the advanced characteristics of this process for preparation of nanosized drug particles^3,6,9–12^. The use of superficial CO_2_ as the secure solvent in pharmaceutical has been accredited by authorities. Moreover, there are some advantages of supercritical CO_2_ method like low price, easy operation, and moderate supercritical factor as compared to different gases. Therefore, supercritical CO_2_ is a great choice to be used as green solvent for preparation of nanomedicine in pharmaceutical area^7,13–16^.

Prior to implementing the nanonization process based on supercritical technology, first drug dissolution has to be analyzed in the solvent. Determination of drug solubility in supercritical process can be done via either experimental approach or computational, by which the computational method is more attractive^17,18^. Applying the experimental technique needs extensive time and cost for the analysis, while computational methods can save time and cost of the experiments, and they can be used for interpolation and extrapolation of the data^19–23^.

Different computational techniques have been utilized for the modeling of drug solubility, but the thermodynamic model and machine learning have shown better performance. The thermodynamic model establishes equilibrium between the solid phase and the solvent phase to determine the value of solubility^19,24–28^. On the other hand, machine learning models need measured data for training and validation of the algorithms^29,30^. The methods of machine learning have shown to be easier to implement and offer better accuracy for prediction of drug solubility in solvents.

Machine learning (ML) as a subject in artificial intelligence is a set of techniques to understand the patterns of data with no any suppostions regarding to the structure. One of these strategies' strengths is creating a relation among data and, then estimate some interaction. An important application of machine learning is regression, that could be defined as a specific type of problem in this study^31–34^. In this research, three approaches have been chose to make approaches on the drug solubility. Accepted methods in this research are Kernel Ridge Regression (KRR), Decision Tree Regression (DTR), and Gaussian Process Regression (GPR). Indeed, we implemented these efficient ML models for the first time for simulation of decitabine solubility in supercritical CO_2_ as the solvent. The results can help to assess the applicability of supercritical process for this drug candidate to be prepared in nanosized scale^35^.

Ridge regressions and the kernel approach are used in the Kernel Ridge Regression (KRR). KRR has the advantage of capturing nonlinear relationships, avoiding regression over-fitting problems through regularization and kernel techniques^35^.

A decision tree regressor (DTR) is a straightforward, comprehensible, and efficient approach. The core principle of the decision tree algorithm has been distributed a large problem within multiple smaller sub-problems, it can be lead to an easier-to-interpret respond^36,37^. A DTR demonstrates a set of conditional queries ordered hierarchically and requested from the tree's root to the leaf^38^. DTRs are easy to understand and have a clear structure. DTRs produce a trained predictor, be able to express principles, and   forecast new datasets using the splitting procedures, which is repeated^39,40^.

The other employed model of this study is based on the Gaussian process (GP) statistical concept, which refers to a group of random variables, as some of them are distributed with Gaussian distributions^41,42^. In geostatistics, the Gaussian process is the fundamental stochastic process. Gaussian processes directly represent Gaussian data and the base for non-Gaussian models such as linear regression models. As a result, Gaussian processes regression based on GPs is both accurate and straightforward for small datasets with high generality^43–45^. Additional target prediction studies of decitabine will be conducted in this research work to get better insights about the different plausible targets for this drug. We will use a hybrid approach in this study through combining both binding and ligand similarity analysis to predict other putative targets of decitabine. The purpose of this research is modifying the solubility of decitabine and GPR has been selected as the best model and reveals that increasing both inputs roughly increase the solubility of drug. So, the optimal is (P = 400, T = 3.38E + 02, Y = 1.07E-03).

## Data set

To make models on solubility, we used a dataset with 32 data points identical to the reference^46,47^. Indeed, the experimental data have been collected from the reference and the machine learning models were fitted and implemented on the data. More detailed description of the method and experimental conditions can be found in the source published paper in^46^. Here, two inputs are considered, Pressure (bar) and Temperature (K), and a single output that shows the solubility of Decitabine drug in the supercritical carbon dioxide (CO_2_). The entire dataset is shown in Table 1.

**Table 1:** The whole dataset^46,47^.

No	Pressure (bar)	Temperature (K)	Y (solubility, mole fraction)
1	120	3.08 × 10^2^	5.04 × 10^–5^
2	120	3.18 × 10^2^	4.51 × 10^–5^
3	120	3.28 × 10^2^	3.69 × 10^–5^
4	120	3.38 × 10^2^	2.84 × 10^–5^
5	160	3.08 × 10^2^	8.23 × 10^–5^
6	160	3.18 × 10^2^	9.37 × 10^–5^
7	160	3.28 × 10^2^	9.11 × 10^–5^
8	160	3.38 × 10^2^	7.79 × 10^–5^
9	200	3.08 × 10^2^	1.18 × 10^–4^
10	200	3.18 × 10^2^	1.55 × 10^–4^
11	200	3.28 × 10^2^	1.77 × 10^–4^
12	200	3.38 × 10^2^	2.05 × 10^–4^
13	240	3.08 × 10^2^	1.37 × 10^–4^
14	240	3.18 × 10^2^	1.87 × 10^–4^
15	240	3.28 × 10^2^	2.82 × 10^–4^
16	240	3.38 × 10^2^	3.71 × 10^–4^
17	280	3.08 × 10^2^	1.76 × 10^–4^
18	280	3.18 × 10^2^	2.40 × 10^–4^
19	280	3.28 × 10^2^	3.42 × 10^–4^
20	280	3.38 × 10^2^	4.90 × 10^–4^
21	320	3.08 × 10^2^	1.97 × 10^–4^
22	320	3.18 × 10^2^	2.69 × 10^–4^
23	320	3.28 × 10^2^	4.27 × 10^–4^
24	320	3.38 × 10^2^	7.15 × 10^–4^
25	360	3.08 × 10^2^	2.18 × 10^–4^
26	360	3.18 × 10^2^	3.40 × 10^–4^
27	360	3.28 × 10^2^	5.60 × 10^–4^
28	360	3.38 × 10^2^	8.74 × 10^–4^
29	400	3.08 × 10^2^	2.83 × 10^–4^
30	400	3.18 × 10^2^	5.06 × 10^–4^
31	400	3.28 × 10^2^	7.88 × 10^–4^
32	400	3.38 × 10^2^	1.07 × 10^–3^

## Methodology

### Kernel ridge regression (KRR)

The first machine learning (ML) method which is considered here for correlation of drug solubility values is the method of Kernel Ridge Regression (KRR). Suppose a data set ${\left\{({x}_{i},{y}_{i})\right\}}_{i=1}^{N}$ has been provided which is include $N$ data points, and the goal is to estimate a function can analysis the mean squared error (MSE) of  [${(f\left(x\right)-y)}^{2}$]. The conditional mean ${f}^{*}\left(x\right) : ={\mathbb{E}}[Y|X=x]$ has been illustrated as the best function^48^. In order to estimate the function ${f}^{*}$,

1 $$\widehat{f}:=\underset{f\in H}{\mathrm{argmin}}\left\{\frac{1}{N}\sum_{i=1}^{N}{\left(f\left({x}_{i}\right)-{y}_{i}\right)}^{2}+ \leftthreetimes {\left|\left|f\right|\right|}_{H}^{2}\right\}$$

This equation can predict the kernel ridge regression^35^.

### Decision tree regression (DTR)

A regression tree or decision trees regressor^38^ uses data from simulation inputs and outputs to create a structure that can be a leaf (terminal node), illustrating a estimation value, or an internal node (decision node), indicating some query to be performed on an input, with a branch and child for each possible output of the query. For continuous inputs, two options are available based on whether the condition is true or not. The structure of the data is declared at every node of the regression tree. To estimate the output for an unobserved data point, the inputs of that data point are employed to traverse the decision tree until a terminal node is seen. The estimated value is decided according to the output values from the training set ending up at that terminal node^51^.

An impurity measure for each node of the tree's test is decided by reviewing all input feature and obtaining an optimal split that maximizes the measure. MSE can be calculated by formulating the split A as follows for a particular input^52^:

2 $$MSE\left(A\right)={p}_{L}.s\left({t}_{L}\right)+{p}_{R}.s\left({t}_{R}\right)$$

Here, t_L_ and tR denote the set of instances. Also, *s*(*t*) indicates the standard deviation of the *N(t)* data, *c*_*i*_*,* of instances within *t*:

3 $$s(t)=\sqrt{\frac{1}{N(t)}\sum_{i=1}^{N(t)}\left({c}_{i}-{\overline{c\left(t\right)}}^{2}\right)}$$

Here, $\overline{c(t)}$ is the average of the values in *t*. The split that minimizes mean square error across all input features for instances at each node of the regression tree is used at each node. Overfitting can occur in tree-based algorithms if the data is split too finely^53,54,52^.

### Gaussian process regression (GPR)

Successor models, such as the Gaussian process (GP), provide predictions as well as the degree of uncertainty associated with those predictions. A GP is a group of random variables with the same Gaussian distribution for any quantity of variables^42^. GPs can be assumed as an infinite-dimensional buildup of multivariate Gaussian distributions. N- instance training data can be considered a singular data point taken from an N-variate Gaussian distribution; thus, it can be matched to the Gaussian process. Typically, the average of this Gaussian Process is reserved to zero.

We describe GP^55^ using a one-dimensional problem with an N-instance training set, [x_i_ | i = 1,2,…,N] and the corresponding output values y = [y_1_, …, y_N_]. We use the same notation as in the previous sections to describe GP for a one-dimensional problem for ease of exposition. Two instances x_i_ and x_j_ in the training set are related to each other through the covariance function k(x_i_, x_j_). The squared-exponentiation function is employed here^56^:

4 $$k\left({x}_{i}, {x}_{j}\right)={\sigma }_{f}^{2}\mathrm{exp}\left(\frac{-{\left({x}_{i}, {x}_{j}\right)}^{2}}{2{l}^{2}}\right)$$

where ${\sigma }_{f}^{2}$ the maximum allowable covariance and *l* is a length parameter that controls the extent of influence of each point. ${\sigma }_{f}^{2}$ Should be set to a large value for functions covering a broad range of values. In condition that data points x_i_ and x_j_ are close to each other, their output values are highly correlated, but if they are far away, then the value at one point does not influence the value at the other point. Accordingly, the hyper-parameter *l* determines the smoothness of the interpolation.

Assume we desire to employ the training data to estimate the output at an unseen data point x_∗_. Since the results be able toe depicted as an instance through a multivariate Gaussian distribution:

5 $$\left[\genfrac{}{}{0pt}{}{y}{{y}_{*}}\right]=\mathrm{ N}\left(0, \left[\genfrac{}{}{0pt}{}{K}{{K}_{*}}\genfrac{}{}{0pt}{}{{K}_{*}^{T}}{{K}_{**}}\right]\right)$$

y denotes the output variable correlated to the N training data points, y_∗_ shows the estimated production at x_∗_ and the following sub-matrices:

6 $$K=\left[\begin{array}{llll}k\left({x}_{1},{x}_{1}\right)& k\left({x}_{1},{x}_{2}\right)& \cdots & k\left({x}_{1},{x}_{N}\right)\\ .& .& .& .\\ \vdots & \vdots & \vdots & \vdots \\ k\left({x}_{N},{x}_{1}\right)& k\left({x}_{N},{x}_{2}\right)& \cdots & k\left({x}_{N},{x}_{3}\right)\end{array}\right]$$

And:

$${K}_{*}=\left[\begin{array}{llll}k({x}_{*},{x}_{1})& & \cdots & k({x}_{*},{x}_{N})\end{array}\right]$$

The probability of y_∗_, which is, the output at a data point, is formulates as:

$${k}_{**}=k({x}_{*},{x}_{*})$$

The variance indicates the degree of uncertainty in the estimate:

$$\mathrm{var}({y}_{*})={K}_{**}-{K}_{*}{K}^{-1}{K}_{*}^{T}$$

The parameters *l* and σ_f_ of the Gaussian process regressor can be computed from the training subset using a maximum likelihood method. It is also feasible to incorporate a Gaussian noise component in the output variable, however we have supposed that the noise is zero in our current research.

### Prediction of decitabine putative targets

Decitabine Smiles were generated via PubChem (https://pubchem.ncbi.nlm.nih.gov/compound/Decitabine) then we feed the smiles into The LigTMap server (https://cbbio.online/LigTMap/?action=home) to identify the plausible targets from seventeen target classes and more than six thousands of different types of proteins.

## Results and discussion

### Analysis of model outcomes

The three abovementioned models were implemented to the collected dataset to build the models for the drug solubility. The hyper-parameters of the models we introduced were optimized using Grid-Search^57^. More than 1000 distinct combinations were used to get these ideal parameters for each model. Then, the models were tested in their ideal configurations, and their performance was evaluated.

Three traditional statistical metrics will be used to assess and compare the efficiency of each model, such as R^2^ and Mean Absolute Error (MAE) and MAPE. In order to calculate each of the statistics, a mathematical equation must be used^52^:

7 $${R}^{2}=\frac{{({\sum }_{i=1}^{\text{n}}({Y}_{i,m}-{\overline{Y}}_{i,m})({Y}_{i,o}- {\overline{Y}}_{i,o}))}^{2}}{{\sum }_{i=1}^{\text{n}}{({Y}_{i,m}-{\overline{Y}}_{i,m})}^{2}{\sum }_{i=1}^{\text{n}}{({Y}_{i,o}- {\overline{Y}}_{i,o})}^{2}}$$

8 $$MAE=\frac{1}{n}\sum_{i=1}^{\text{n}}\left|{Y}_{i,m}-{Y}_{\text{i,o}}\right|$$

**Figure 1:**
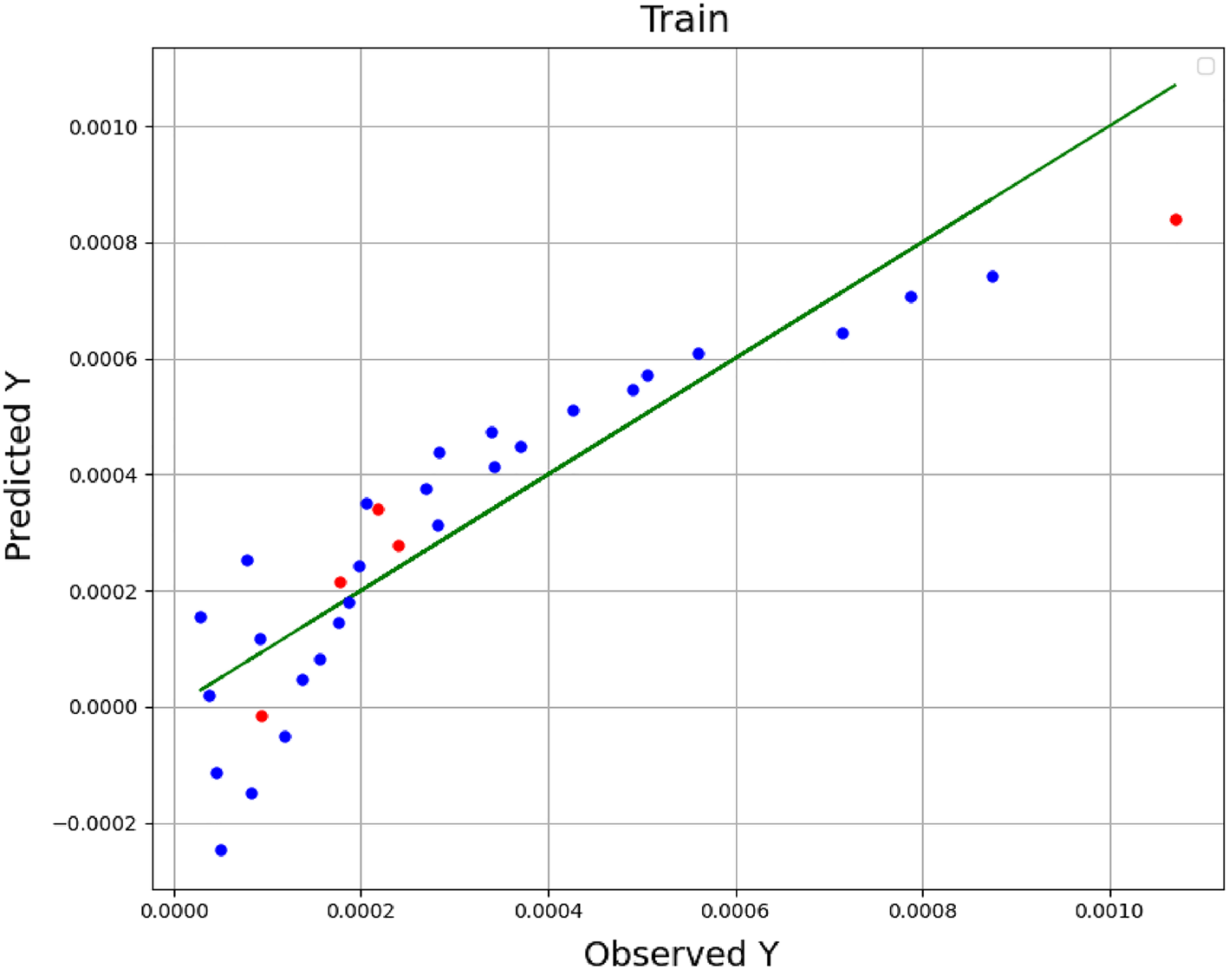
Observed vs estimated values (KRR) (Y: solubility/mole fraction).

**Figure 2:**
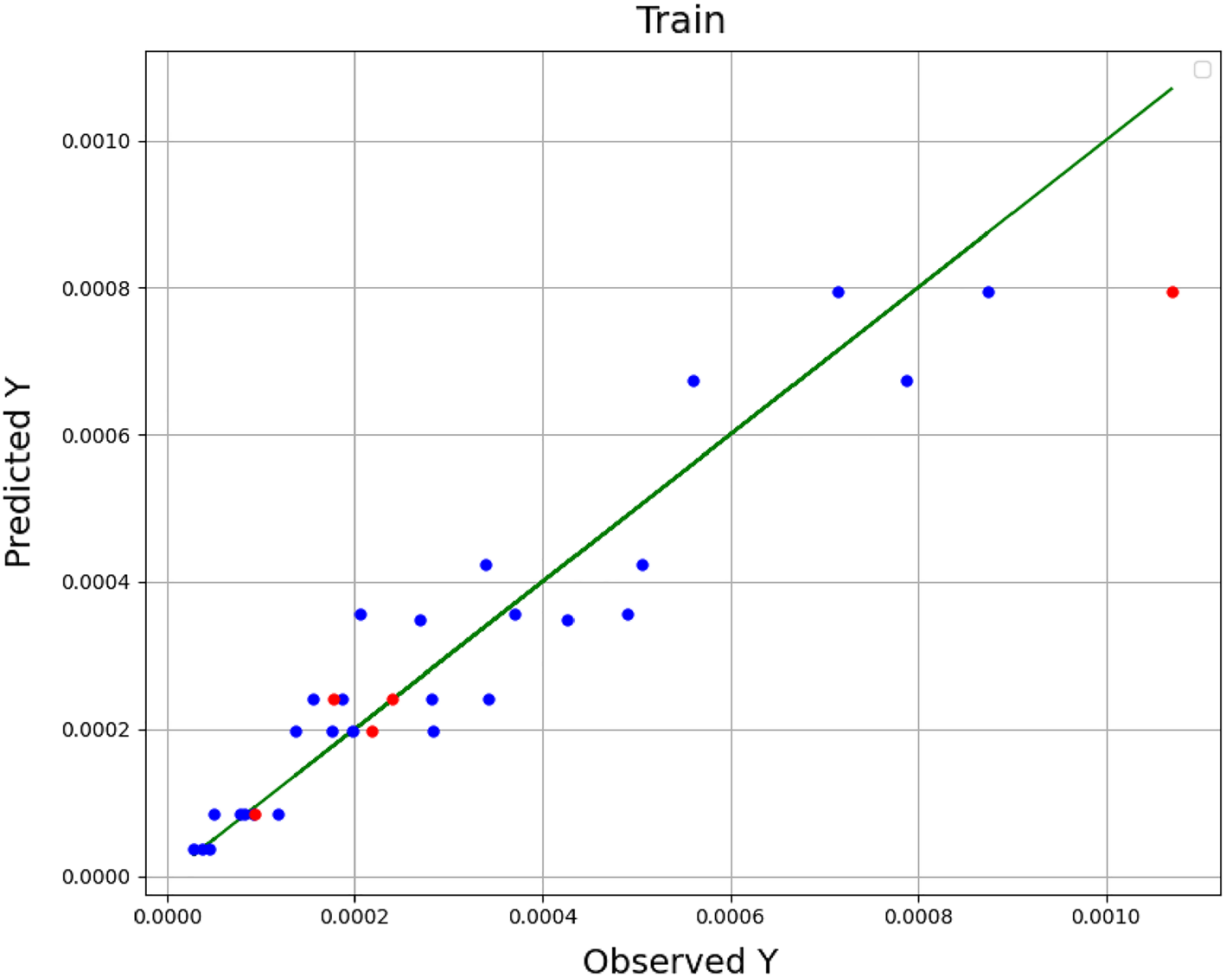
Observed vs estimated values (DTR) (Y: solubility/mole fraction).

**Figure 3:**
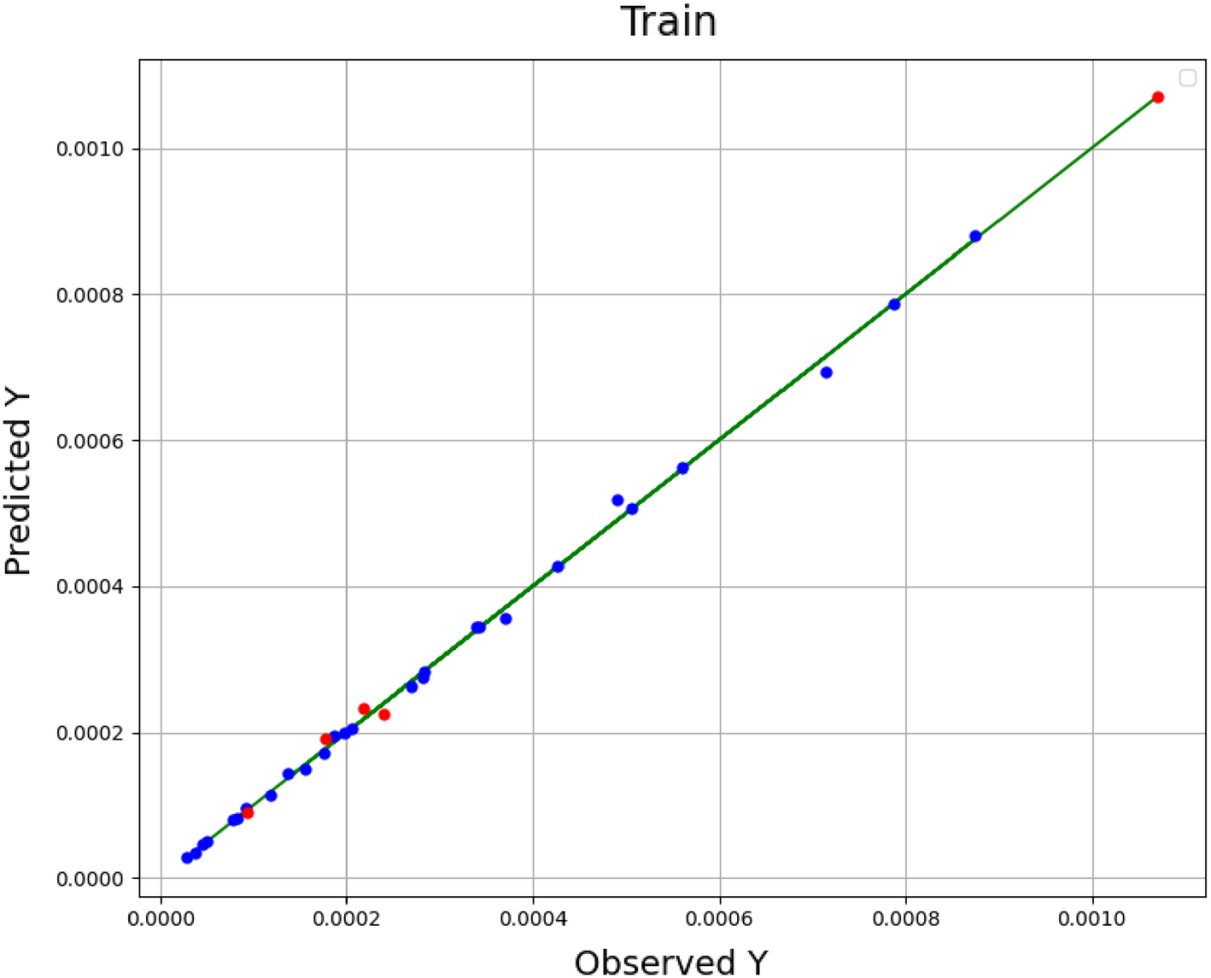
Observed vs predicted values (GPR) (Y: solubility/mole fraction).

In these equations, *n* is size of data set, *Y*_*i,m*_ is the estimated value, *Y*_*i,o*_ indicates actual (observed) value. As well, ${\overline{Y}}_{i,m}$ is the average of estimated values and ${\overline{Y}}_{i,o}$ indicates average of actual values. A comparison among the estimated amounts and the real (observed) amounts in the model training is shown in Figs. 1, 2, and 3 for the methods of KRR, DTR, and GPR, respectively. The red dots indicate the test data, the blue dots are the training data (estimated amounts), and the green line represents the real amounts. Comparing these three shapes clearly shows the higher generality in the GPR method in comparison to other methods. The statistical results of the comparison for all methods have been also demonstrated in Table 2. As it is clear, all methods have great capability in fitting and correlating the experimental data which indicate that these models are of great choice for application in production of nanomedicine using supercritical based technology. The best outputs are illustrated for GPR through R^2^ higher than 0.99 in order to fit the solubility results.

**Table 2:** The statistical results of all models used in this study.

Models	MAE	R^2^	MAPE
KRR	1.08E−04	0.806	4.64E−01
DTR	7.40E−05	0.891	1.63E−01
GPR	9.73E−06	0.998	5.06E−02

**Figure 4:**
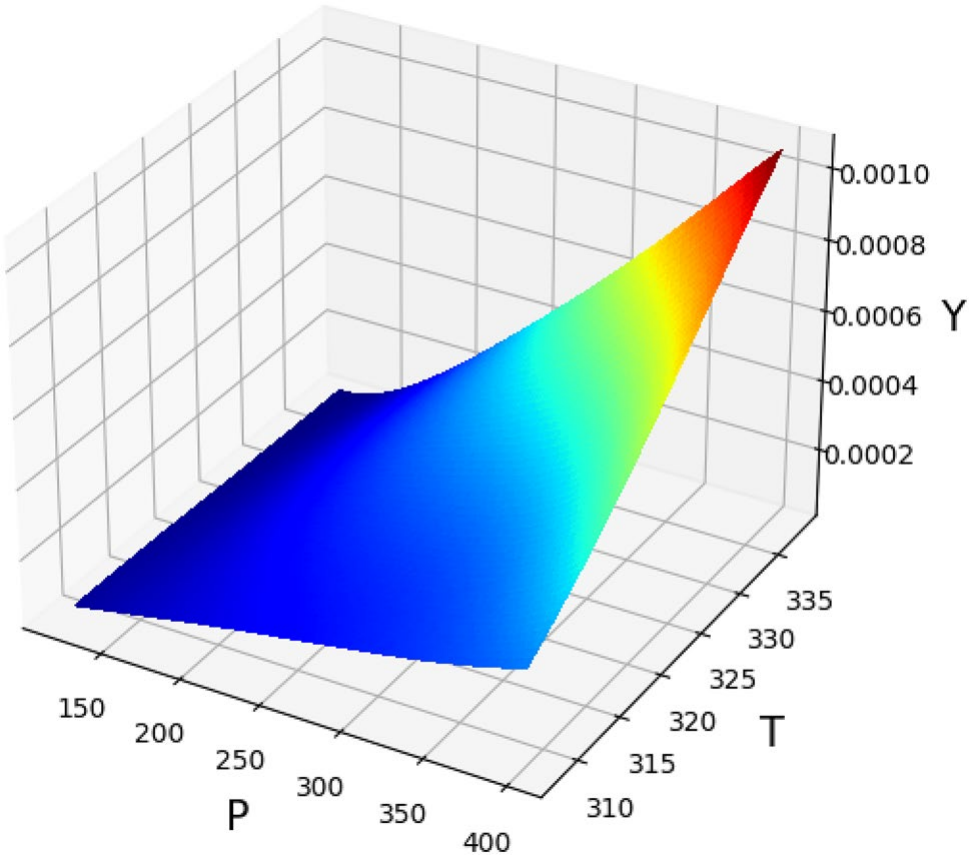
3D projection of inputs/outputs (GPR method) (T: temperature, K), (P: pressure, bar).

**Figure 5:**
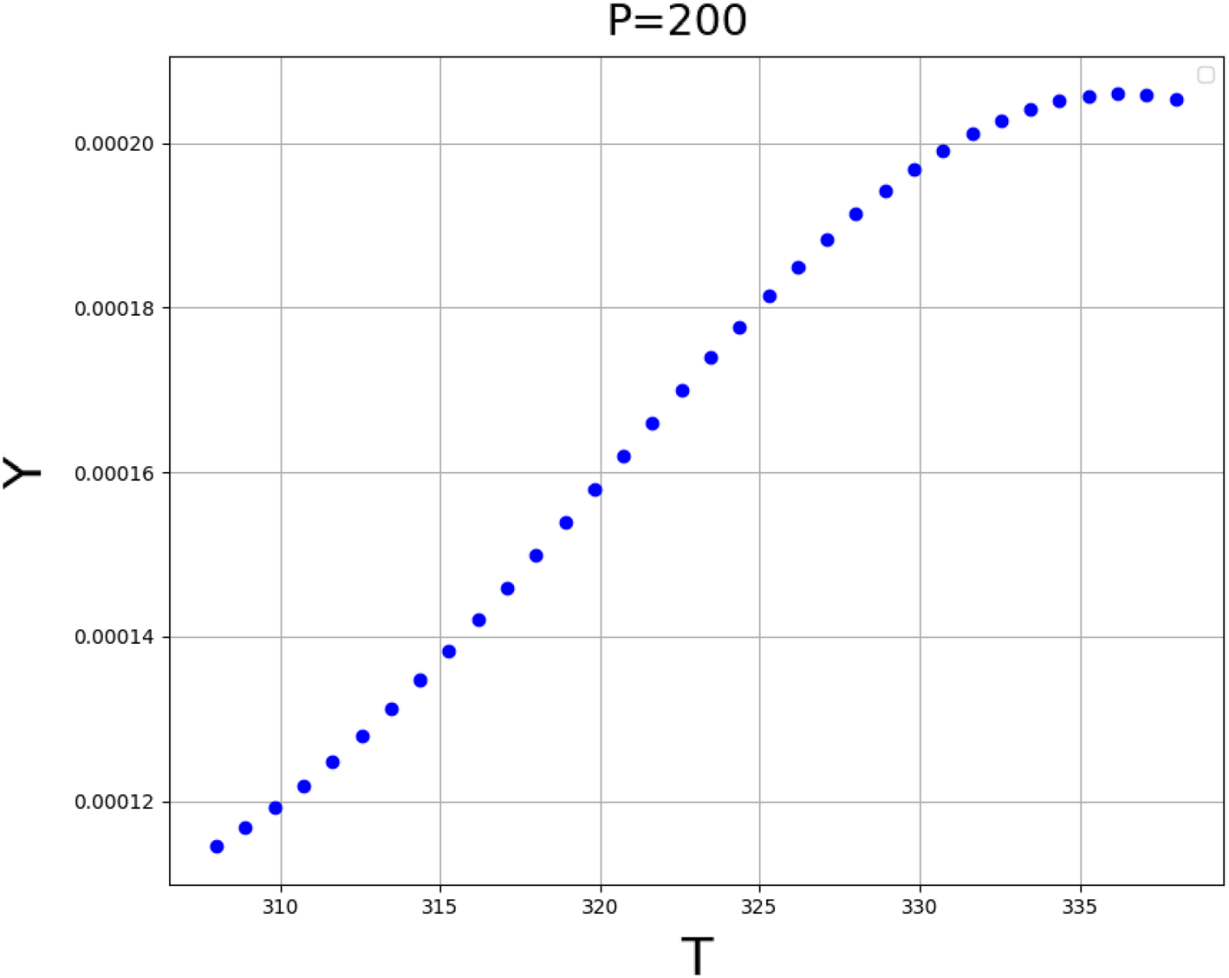
Trend of variable T (temperature, K) calculated using GPR model (Y: solubility, mole fraction).

**Figure 6:**
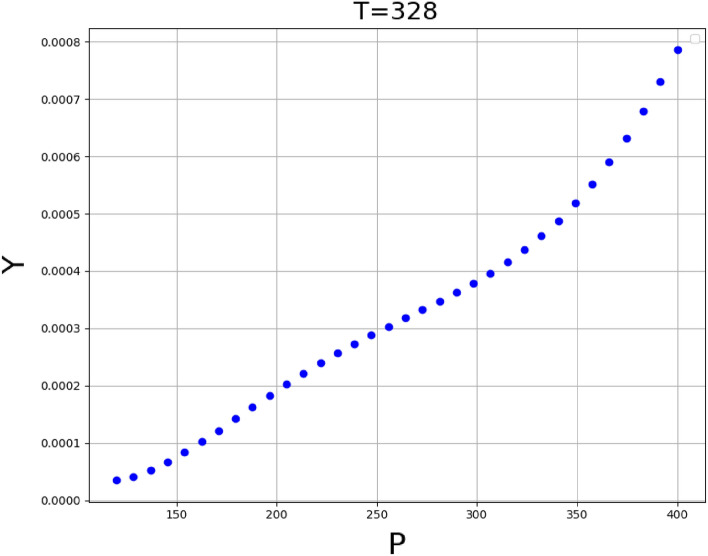
Trend of variable P (pressure, bar) calculated using GPR model (Y: solubility, mole fraction).

The validated GPR method as the significant method has been applied to calculate the solubility data and find the influence of temperature and the pressure on the solubility of Decitabine in supercritical CO_2_. The results of 3D surface plot are explained in Fig. 4, the impact of temperature and pressure on the solubility of decitabine are significant, so that the highest value of solubility is observed at the maximum values of T and P in the 3D graph (see Fig. 4). The increase in the solubility with temperature and pressure could be attributed to the change of solvent density and consequently changing the solvation capacity of the solvent. Also, the 2D graphs of solubility versus temperature and pressure are indicated in Figs. 5 and 6, respectively. The optimum values calculated using the GPR model are listed in Table 3.

**Table 3:** Optimized  parameters using the GPR method.

P (bar)	T (K)	Y (mole fraction)
400.0	338.0	0.001069

**Figure 7:**
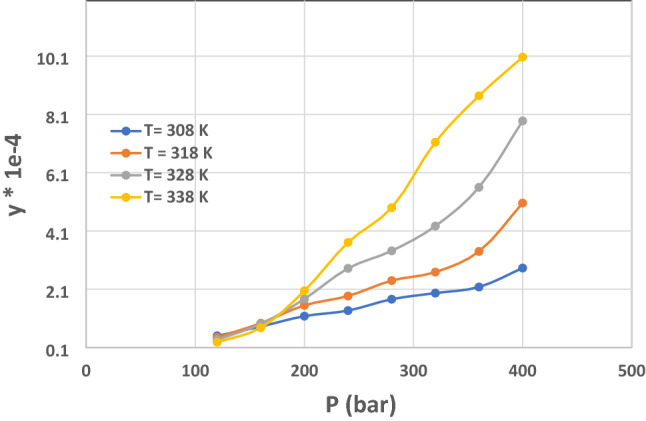
The impact of pressure on the solubility of Decitabine considering disparate temperatures.

Figure 7 aims to evaluate the impact of pressure on the solubility values of Decitabine at disparate temperatures. As indicated, there is a cross over area at each solubility figure. Indeed, the impact of temperature on drug solubility in SC-CO_2_ is paradoxical. Furthur, temperature's growth, influence on the sublimation pressure of drugs, causes the increment of solubility. In another side, increase the temperature results in decreasing the molecular compaction and as the result, the amount of SC-CO2 density, which has negative effect on the solubility of Decitabine. The pressure value of 18 bar is known as the cross over pressure. At the pressures between 12 and 18 bar, the negative effect of density deterioration entirely overcomes the desirable effect of vapor pressure increment. Moreover, at this range of pressures, temperature enhancement lead to a reduction in solubility. Above the cross over pressure (18 MPa), the solubility of Decitabine significantly enhances owing to the superiority of the positive effect of drug’s vapor pressure than the negative effect of density reduction. Therefore, at this pressure elevation of temperature improves the solubility.

### Correlation of the solubility data with semi-empirical models

**Figure 8:**
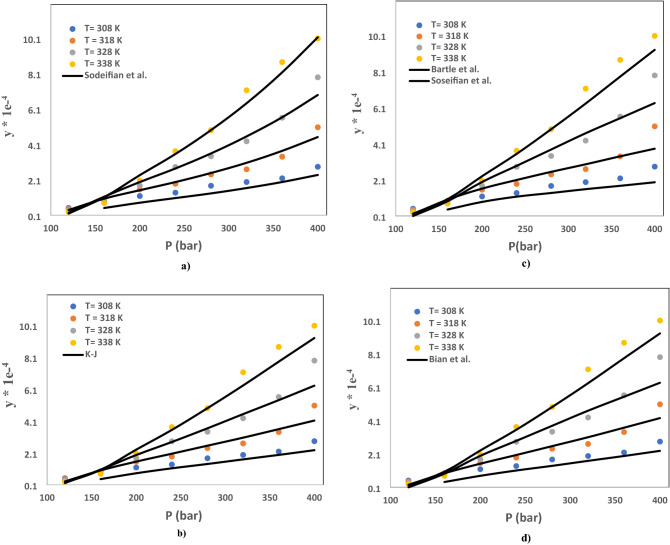
Comparison of correlation outcomes for Decitabine-SC-CO_2_ system using various semi-empirical models. (**a**) Sodeifian et al. (**b**) K-J, (**c**) Bartle et al. and (**d**) Bian et al.

Figure 8a–d present the correlation outcomes of Decitabine-SC-CO_2_ system obtained by semi-empirical models. In this investigation, four principal semi-empirical density-based models (Sodeifian et al., K-J, Bartle et al. and Bian et al.) were pondered for the correlation of the experimental data of Decitabine solubility SC-CO_2_^58–62^. Disparate values including settable parameters (a_0_, a_1_, a_2_, a_3_, _a4_ and a_5_), average absolute relative deviation (AARD%) and R^2^ are enlisted in Table 4. The AARD for developed models for Sodeifian et al., Bartle et al. and Bian et al. models were 12.15%, 11.61%, 14.46%, and 13.25%, respectively. Comparison of the results implies the fact that K-J model is the best model due to presenting the lowest value of AARD (11.61%).

**Table 4:** Correlation outcomes of Decitabine-SC-CO_2_ system obtained by various semi-empirical models.

Model	a_0_	a_1_	a_2_	a_3_	a_4_	a_5_	AARD	R^2^
Sodeifian et al	−69.709	0.03619	5.8489	−2.8E−05	0.003501	−583.369	12.15	0.9806
K-J	7.32	−7108.7	0.00769	–	–	–	11.61	0.9641
Bartle et al	25.15	−9537.9	0.0122	–	–	–	14.46	0.9520
Bian et al	2.2205	0.00083	−7089.51	−0.2957	−5.197	–	13.25	0.9703

Table 4 presents the correlation outcomes of Decitabine-SC-CO_2_ system obtained by semi-empirical models.

**Figure 9:**
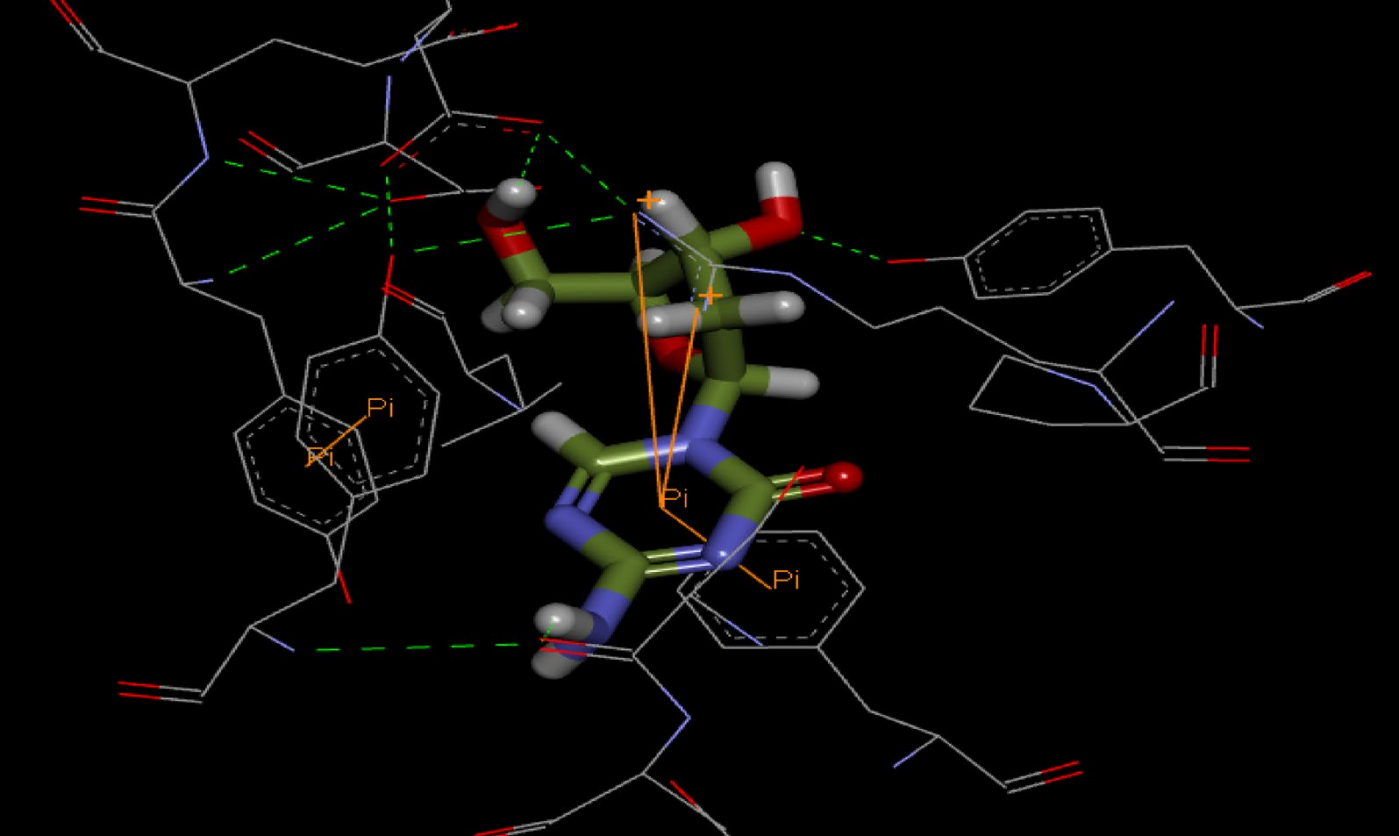
3D interactions and binding mode of decitabine drug with Thymidylate kinase (Pdb ID: 1mrs).

Additionally, through usage of LigTMap server we have found more than one hundred predicted targets for decitabine, these targets are classified according to disease target class into kinase 34 (29%), 30 (25%) transferase, 28 (24%) Hydrolase, 15 (13%) tuberculosis, 5 (4%) Hpyroli, 3 (2.5%) Influenza and 1 (0.8%) Beta secretase. Attached with this research work a supplementary data file that contains a list for the targets with docking scores in the binding sites of the specified proteins. Also, Pdb IDs for each specific protein are incorporated, the optimum binding of decitabine with these target proteins and binding mode, in addition to predicted affinity and docking scores all are obtained through the automated workflow of LigTMap. The obtained results (supplementary data) revealed that decitabine has ligand Similarity Score more than 0.6 with Deoxycytidine kinase and Thymidylate kinase TMK, target classes are kinase and tuberculosis with Pdb ID 3ipx and 1w2g respectively. Decitabine showed binding affinity more than 7 in disease target class Influenza, target name is Polymerase basic protein 2 (Pdb IDs: 5efc, 4or6 and 4q46). Decitabine showed the best docking score into Thymidylate kinase binding site with value equal −7.007 kcal/mol, Pdb ID: 1mrs in tuberculosis disease class. From these obtained results we can conclude that Thymidylate kinase (tuberculosis) and Polymerase basic protein 2 (Influenza) are plausible targets for decitabine, the following Fig. 9 illustrates the binding mode with Thymidylate kinase (tuberculosis).

Four semi-empirical models (Sodeifian et al., Bartle et al., K-J and Bian et al.) have been considered to make a correlation with the outputs of solubility experiments. The precision for all applied methods has analysed and measured through AARD% and R^2^. Comparison of the outputs implies the fact that K-J model is the best model due to presenting the lowest value of AARD (11.61%). Despite good efficiency of K-J model for the accurate prediction of drug solubility, the employed GPR model shows better performance compared to K-J owing to having higher value of R^2^.

## Conclusion

Computational simulation of Decitabine drug solubility in supercritical carbon dioxide was carried out in this study via three different machine learning models. We used a dataset of 32 data points and two inputs in this investigation to create solubility models (P and T). In this dataset, Y (solubility, mole fraction) is the lone result which is predicted by the models. Kernel Ridge Regression (KRR), Decision Tree Regression (DTR), and Gaussian process (GP) are the models which were employed in this work for correlation of the solubility data. Hyper-parameter tweaking was used to fine-tune these models, and standard metrics were used to assess their performance. KRR, DTR, and GPR have R^2^-scores of 0.806, 0.891, and 0.998. MAE's error rate is 1.08E−04, 7.40E−05, and 9.73E−06 in that sequence, too. The MAPE measure has a KRR error rate of 4.64E−01, a DTR error rate of 1.63E−01, and a GPR error rate of 5.06E−02 as the optimum option. As a conclusion, the best model (GPR) shows that increasing both inputs roughly raise the output. So, the best outcome is obtained as P = 400 bar, T = 3.38E + 02 °K, Y = 1.07E−03. Finally, LigTMap workflow revealed the promiscuity of decitabine to target Thymidylate kinase (disease class: tuberculosis) and Polymerase basic protein 2 (disease class: influenza). In this paper, the solubility value of Decitabine was evaluated at disparate values of pressure (120, 160, 200, 240, 280, 320, 360 and 400 bar) and temperatures (308, 318, 328, and 338 K). Four semi-empirical models (Sodeifian et al., Bartle et al., K-J and Bian et al.) have been considered to make a correlation with the outputs of solubility experiments. The precision of all applied methods has been evaluated through AARD% and R^2^. Comparison of the outputs implies the fact that K-J model is the best model due to presenting the lowest value of AARD (11.61%). Despite good efficiency of K-J model for the accurate prediction of drug solubility, the employed GPR model shows better performance compared to K-J owing to having higher value of R^2^.

## Supplementary Information

Supplementary Information.

## Data Availability

All data are available within the published paper.

## Abbreviations

ML: Machine learning
P: Pressure
T: Temperature
KRR: Kernel ridge regression
GPR: Gaussian process regression
DTR: Decision tree regression
MSE: Mean squared error
MAE: Mean absolute error
AARD: Average absolute relative deviation
SC-CO_2_: Supercritical CO_2_
