# Supplementary material for: Computational simulation and target prediction studies of solubility optimization of decitabine through supercritical solvent
Source: Sci Rep. 2022 Nov 7;12:18875. doi: 10.1038/s41598-022-21233-0 (PMC9640585; doi:10.1038/s41598-022-21233-0)
Supplement: Supplementary file 1 — Supplementary Information. [file 41598_2022_21233_MOESM1_ESM.pdf]

Result of Job ID #ackvz

Selected target classes: Anticogulant,Beta_secretase,Bromodomain,Carbonic_Anhydrase,Estrogen,Hydrolase,Isomerase,Kinase,Ligase,Peroxisome,Transferase,Diabetes,HCV,Hpyroli,HIV,Influenza,Tuberculosis
Description: (No description)
Cutoff: 0.4

Input SMILES: C1C(C(OC1N2C=NC(=NC2=O)N)CO)O

No. of targets found for selected target classes

Total	Kinase	Transferase	Hydrolase	Tuberculosis	Hpyroli	Influenza	Beta_secretas
116	34	30	28	15	5	3	1

Target list CSV

Rank	PDB	Target Class	Target Name	Ligand Name	Ligand Similarity Score	Binding Similarity Score	LigTMap Score	Predicted Affinity (-log M)	PSOVina2 Docking Score (kcal/mol)	PSOVina2 Docking Pose	Selec bind simil anal
1	3ipx	Kinase	Deoxycytidine kinase	B86	0.619	0.462	0.572	5.702	-6.071	3D PDB	Do
2	1w2g	Tuberculosis	THYMIDYLATE KINASE TMK	THM	0.642	0.2	0.509	4.685	-6.658	3D PDB	Do
3	5dv2	Hydrolase	CCR4-NOT transcription complex subunit 6-like	C5P	0.472	0.583	0.506	6.004	-6.016	3D PDB	Do
4	5edd	Tuberculosis	Deoxyuridine 5'-triphosphate nucleotidohydrolase	DUP	0.508	0.455	0.492	4.685	-4.435	3D PDB	Do
4	4kc2	Transferase	Fucosylglycoprotein alpha-N-acetylgalactosaminyltransferase soluble form	WS2	0.472	0.538	0.492	4.599	-6.528	3D PDB	Do

Rank	PDB	Target Class	Target Name	Ligand Name	Ligand Similarity Score	Binding Similarity Score	LigTMap Score	Predicted Affinity (-log M)	PSOVina2 Docking Score (kcal/mol)	PSOVina2 Docking Pose	Selec bind simil anal
5	4nxu	Transferase	Mitochondrial dynamic protein MID51	ADP	0.43	0.625	0.488	4.599	-5.755	3D PDB	Do
6	1mrs	Tuberculosis	THYMIDYLATE KINASE	5HU	0.54	0.333	0.478	4.685	-7.007	3D PDB	Do
7	2py4	Tuberculosis	Deoxyuridine 5'-triphosphate nucleotidohydrolase	DUP	0.508	0.4	0.476	4.685	-4.475	3D PDB	Do
8	5eom	Kinase	Protein mab-21-like 1	CTP	0.454	n.a.	0.454	5.702	-5.353	3D PDB	Do
9	1y8p	Kinase	[Pyruvate dehydrogenase [lipoamide]] kinase isozyme 3	ATP	0.422	0.467	0.435	5.702	-6.398	3D PDB	Do
10	1mrn	Tuberculosis	Thymidylate Kinase	T5A	0.439	0.417	0.433	4.685	-6.453	3D PDB	Do
11	4gz3	Transferase	UDP-N-acetylglucosamine--peptide N-acetylglucosaminyltransferase 110 kDa subunit	UDP	0.466	0.353	0.432	4.599	-5.433	3D PDB	Do
12	3ioe	Tuberculosis	Pantothenate synthetase	A7D	0.455	0.375	0.431	4.685	-6.554	3D PDB	Do
13	4u0u	Transferase	Adenosine monophosphate-protein transferase FICD	ADP	0.43	n.a.	0.43	4.599	-5.411	3D PDB	Do
14	4kc4	Transferase	Fucosylglycoprotein alpha-N-acetylgalactosaminyltransferase	WS3	0.472	0.308	0.423	4.599	-6.052	3D PDB	Do
14	2zoq	Kinase	Mitogen-activated protein kinase 3	5ID	0.475	0.3	0.423	5.702	-6.41	3D PDB	Do
15	3v0p	Transferase	Histo-blood group ABO system transferase	4GW	0.419	0.429	0.422	4.599	-6.588	3D PDB	Do
16	3d2r	Kinase	[Pyruvate dehydrogenase [lipoamide]] kinase isozyme 4	ADP	0.43	0.4	0.421	5.702	-6.42	3D PDB	Do
16	4i3z	Kinase	Cyclin-dependent kinase 2	ADP	0.43	0.4	0.421	5.702	-6.09	3D PDB	Do
17	5efc	Influenza	Polymerase basic protein 2	GTP	0.416	0.429	0.42	7.773	-4.839	3D PDB	Do

Rank	PDB	Target Class	Target Name	Ligand Name	Ligand Similarity Score	Binding Similarity Score	LigTMap Score	Predicted Affinity (-log M)	PSOVina2 Docking Score (kcal/mol)	PSOVina2 Docking Pose	Selec bind simil anal
18	3iob	Tuberculosis	Pantothenate synthetase	A4D	0.468	0.3	0.418	4.685	-6.233	3D PDB	Do
19	2y8q	Kinase	5'-AMP-ACTIVATED PROTEIN KINASE CATALYTIC SUBUNIT ALPHA-1	ADP	0.43	0.385	0.416	5.702	-5.599	3D PDB	Do
20	1ryh	Hydrolase	ras-related C3 botulinum toxin substrate 1 isoform Rac1b	GNP	0.439	0.357	0.414	6.004	-4.634	3D PDB	Do
21	3hb8	Transferase	Thymidylate synthase	UMP	0.554	0.083	0.413	4.599	-5.036	3D PDB	Do
21	3nyn	Kinase	G protein-coupled receptor kinase 6	SGV	0.482	0.25	0.413	5.702	-6.271	3D PDB	Do
22	5ect	Tuberculosis	Deoxyuridine 5'-triphosphate nucleotidohydrolase	DUP	0.508	0.182	0.41	4.685	-4.544	3D PDB	Do
23	4y64	Transferase	Histo-blood group ABO system transferase	48C	0.477	0.25	0.409	4.599	-6.206	3D PDB	Do
23	5i2f	Hydrolase	Histidine triad nucleotide-binding protein 1	BS5	0.412	0.4	0.409	6.004	-5.387	3D PDB	Do
24	4rcg	Tuberculosis	Phosphoenolpyruvate carboxykinase [GTP]	GDP	0.424	0.364	0.406	4.685	-5.784	3D PDB	Do
25	4ay5	Transferase	UDP-N-ACETYLGLUCOSAMINE--PEPTIDE N-ACETYLGLUCOSAMINYL TRANSFERASE 110 KDA SUBUNIT	UDP	0.466	0.263	0.405	4.599	-5.772	3D PDB	Do
26	3h30	Kinase	Casein kinase II subunit alpha	RFZ	0.453	0.286	0.403	5.702	-6.032	3D PDB	Do
27	5ax3	Kinase	Mitogen-activated protein kinase 1	5ID	0.475	0.222	0.399	5.702	-5.75	3D PDB	Do
28	2pze	Hydrolase	Cystic fibrosis transmembrane conductance regulator	ATP	0.422	0.333	0.395	6.004	-4.031	3D PDB	Do
28	4de7	Tuberculosis	GLUCOSYL-3-PHOSPHOGLYCERATE SYNTHASE (GpgS)	UDP	0.479	0.2	0.395	4.685	-5.972	3D PDB	Do
29	2hqu	Hydrolase	Deoxyuridine 5'-triphosphate nucleotidohydrolase	DUP	0.508	0.125	0.393	6.004	-3.744	3D PDB	Do

Rank	PDB	Target Class	Target Name	Ligand Name	Ligand Similarity Score	Binding Similarity Score	LigTMap Score	Predicted Affinity (-log M)	PSOVina2 Docking Score (kcal/mol)	PSOVina2 Docking Pose	Selec bind simil anal
30	3h9k	Transferase	Thymidylate synthase	UFP	0.523	0.083	0.391	4.599	-5.302	3D PDB	Do
31	4kod	Hydrolase	Transitional endoplasmic reticulum ATPase	ADP	0.43	0.273	0.383	6.004	-5.14	3D PDB	Do
32	4ay6	Transferase	UDP-N-ACETYLGLUCOSAMINE--PEPTIDE N-ACETYLGLUCOSAMINYLTRANSFERASE 110 KDA SUBUNIT	12V	0.407	0.318	0.381	4.599	-5.785	3D PDB	Do
33	3ocp	Transferase	PRKG1 protein	CMP	0.434	0.25	0.379	4.599	-5.11	3D PDB	Do
33	3bbf	Kinase	Nucleoside diphosphate kinase B	GDP	0.424	0.273	0.379	5.702	-5.433	3D PDB	Do
34	4kc1	Transferase	Fucosylglycoprotein alpha-N-acetylgalactosaminyltransferase	WS1	0.472	0.154	0.377	4.599	-6.137	3D PDB	Do
35	3p23	Kinase	Serine/threonine-protein kinase/endoribonuclease IRE1	ADP	0.43	0.25	0.376	5.702	-5.874	3D PDB	Do
36	1b38	Kinase	PROTEIN (CELL DIVISION PROTEIN KINASE 2)	ATP	0.422	0.267	0.375	5.702	-6.192	3D PDB	Do
36	1b39	Kinase	PROTEIN (CELL DIVISION PROTEIN KINASE 2)	ATP	0.422	0.267	0.375	5.702	-5.926	3D PDB	Do
37	1w2h	Tuberculosis	THYMIDYLATE KINASE TMK	ATM	0.481	0.125	0.374	4.685	-5.825	3D PDB	Do
37	3q6z	Transferase	Poly [ADP-ribose] polymerase 14	APR	0.406	0.3	0.374	4.599	-6.145	3D PDB	Do
38	4fl2	Kinase	Tyrosine-protein kinase SYK	ANP	0.416	0.273	0.373	5.702	-5.961	3D PDB	Do
39	4or6	Influenza	Polymerase basic protein 2	GDP	0.424	0.25	0.372	7.773	-4.513	3D PDB	Do
40	4wop	Tuberculosis	ATP-dependent dethiobiotin synthetase BioD	CTP	0.454	0.176	0.371	4.685	-5.68	3D PDB	Do
41	1a4r	Hydrolase	G25K GTP-BINDING PROTEIN	GNH	0.451	0.182	0.37	6.004	-5.021	3D PDB	Do

Rank	PDB	Target Class	Target Name	Ligand Name	Ligand Similarity Score	Binding Similarity Score	LigTMap Score	Predicted Affinity (-log M)	PSOVina2 Docking Score (kcal/mol)	PSOVina2 Docking Pose	Selec bind simil anal
41	1pkx	Transferase	Bifunctional purine biosynthesis protein PURH	XMP	0.432	0.227	0.37	4.599	-4.736	3D PDB	Do
41	1ucn	Kinase	nucleoside diphosphate kinase A	ADP	0.43	0.231	0.37	5.702	-5.181	3D PDB	Do
42	5htb	Kinase	Serine/threonine-protein kinase haspin	6L5	0.44	0.2	0.368	5.702	-3.016	3D PDB	Do
43	3my1	Kinase	Cell division protein kinase 9	RFZ	0.453	0.167	0.367	5.702	-5.65	3D PDB	Do
44	4kwp	Kinase	Casein kinase II subunit alpha	EXX	0.523	0	0.366	5.702	-5.726	3D PDB	Do
44	4fl3	Kinase	Tyrosine-protein kinase SYK	ANP	0.416	0.25	0.366	5.702	-5.807	3D PDB	Do
44	1fta	Hydrolase	FRUCTOSE-1,6-BISPHOSPHATASE	AMP	0.437	0.2	0.366	6.004	-6.656	3D PDB	Do
44	3juk	Hpyroli	UDP-glucose pyrophosphorylase (GalU)	UPG	0.441	0.19	0.366	5.28	-5.987	3D PDB	Do
45	3zjc	Hydrolase	GTPASE IMAP FAMILY MEMBER 7	GNP	0.411	0.25	0.362	6.004	-6.336	3D PDB	Do
46	4y63	Transferase	Histo-blood group ABO system transferase	48O	0.449	0.154	0.36	4.599	-6.112	3D PDB	Do
47	1q91	Hydrolase	5(3)-deoxyribonucleotidase	DPB	0.427	0.2	0.359	6.004	-6.866	3D PDB	Do
48	3od0	Transferase	PRKG1 protein	PCG	0.425	0.2	0.358	4.599	-5.489	3D PDB	Do
49	2zkj	Kinase	[Pyruvate dehydrogenase [lipoamide]] kinase isozyme 4	ADP	0.43	0.188	0.357	5.702	-6.617	3D PDB	Do
50	3ehw	Hydrolase	dUTP pyrophosphatase	DUP	0.508	0	0.356	6.004	-3.34	3D PDB	Do
51	5lvn	Kinase	3-phosphoinositide-dependent protein kinase 1	ADN	0.505	0	0.354	5.702	-5.64	3D PDB	Do
51	4ckj	Kinase	PROTO-ONCOGENE TYROSINE-PROTEIN KINASE RECEPTOR RET	ADN	0.505	0	0.354	5.702	-6.58	3D PDB	Do

Rank	PDB	Target Class	Target Name	Ligand Name	Ligand Similarity Score	Binding Similarity Score	LigTMap Score	Predicted Affinity (-log M)	PSOVina2 Docking Score (kcal/mol)	PSOVina2 Docking Pose	Selec bind simil anal
51	4cki	Kinase	PROTO-ONCOGENE TYROSINE-PROTEIN KINASE RECEPTOR RET	ADN	0.505	0	0.354	5.702	-6.094	3D PDB	Do
52	4fvr	Kinase	Tyrosine-protein kinase JAK2	ATP	0.422	0.188	0.351	5.702	-5.991	3D PDB	Do
52	3rw9	Transferase	Spermidine synthase	DSH	0.44	0.143	0.351	4.599	-6.76	3D PDB	Do
53	3my5	Kinase	Cell division protein kinase 2	RFZ	0.453	0.111	0.35	5.702	-5.84	3D PDB	Do
53	4ek9	Transferase	Histone-lysine N-methyltransferase, H3 lysine-79 specific	EP4	0.439	0.143	0.35	4.599	-5.859	3D PDB	Do
54	4lqy	Hydrolase	Bis(5'-adenosyl)-triphosphatase ENPP4	AMP	0.437	0.143	0.349	6.004	-5.06	3D PDB	Do
55	3qxc	Hpyroli	Dethiobiotin synthetase	ATP	0.422	0.176	0.348	5.28	-6.243	3D PDB	Do
55	4kln	Hydrolase	Transitional endoplasmic reticulum ATPase	AGS	0.411	0.2	0.348	6.004	-5.031	3D PDB	Do
55	4ko8	Hydrolase	Transitional endoplasmic reticulum ATPase	AGS	0.411	0.2	0.348	6.004	-4.722	3D PDB	Do
56	4ido	Hydrolase	Atlastin-1	GDP	0.424	0.167	0.347	6.004	-6.108	3D PDB	Do
57	4o1l	Kinase	Adenosine kinase	HO4	0.474	0.048	0.346	5.702	-7	3D PDB	Do
58	4fvq	Kinase	Tyrosine-protein kinase JAK2	ATP	0.422	0.154	0.341	5.702	-6.067	3D PDB	Do
58	3mle	Hpyroli	Dethiobiotin synthetase	ADP	0.43	0.133	0.341	5.28	-5.637	3D PDB	Do
59	4pvv	Tuberculosis	Adenosine kinase	HO4	0.408	0.182	0.34	4.685	-5.314	3D PDB	Do
59	3q71	Transferase	Poly [ADP-ribose] polymerase 14	AR6	0.406	0.188	0.34	4.599	-6.274	3D PDB	Do
60	2i6a	Kinase	Adenosine kinase	5I5	0.403	0.182	0.336	5.702	-6.752	3D PDB	Do

Rank	PDB	Target Class	Target Name	Ligand Name	Ligand Similarity Score	Binding Similarity Score	LigTMap Score	Predicted Affinity (-log M)	PSOVina2 Docking Score (kcal/mol)	PSOVina2 Docking Pose	Selec bind simil anal
60	1ryf	Hydrolase	ras-related C3 botulinum toxin substrate 1 isoform Rac1b	GDP	0.455	0.059	0.336	6.004	-4.656	3D PDB	Do
60	1clu	Hydrolase	TRANSFORMING PROTEIN P21/H-RAS-1	DBG	0.429	0.118	0.336	6.004	-5.769	3D PDB	Do
61	1hi4	Hydrolase	EOSINOPHIL-DERIVED NEUROTOXIN	A3P	0.43	0.111	0.335	6.004	-4.795	3D PDB	Do
62	2ou7	Kinase	Serine/threonine-protein kinase PLK1	ANP	0.416	0.143	0.334	5.702	-5.321	3D PDB	Do
63	4b1j	Hydrolase	POLY(ADP-RIBOSE) GLYCOHYDROLASE	A1R	0.428	0.111	0.333	6.004	-6.343	3D PDB	Do
63	1xsc	Hydrolase	Bis(5'-nucleosyl)-tetraphosphatase	ATP	0.422	0.125	0.333	6.004	-5.301	3D PDB	Do
64	4ekg	Transferase	Histone-lysine N-methyltransferase, H3 lysine-79 specific	0QJ	0.409	0.154	0.332	4.599	-6.094	3D PDB	Do
64	1hi3	Hydrolase	EOSINOPHIL-DERIVED NEUROTOXIN	A2P	0.426	0.111	0.332	6.004	-4.673	3D PDB	Do
65	2c02	Hydrolase	NONSECRETORY RIBONUCLEASE	ADP	0.43	0.091	0.328	6.004	-4.918	3D PDB	Do
66	3v0l	Transferase	Histo-blood group ABO system transferase	2GW	0.417	0.118	0.327	4.599	-4.788	3D PDB	Do
67	1hi5	Hydrolase	EOSINOPHIL-DERIVED NEUROTOXIN	ADP	0.43	0.083	0.326	6.004	-4.99	3D PDB	Do
68	2kmx	Hydrolase	Copper-transporting ATPase 1	ATP	0.422	0.1	0.325	6.004	-5.561	3D PDB	Do
69	4idn	Hydrolase	Atlastin-1	GNP	0.411	0.118	0.323	6.004	-6.134	3D PDB	Do
70	4eki	Transferase	Histone-lysine N-methyltransferase, H3 lysine-79 specific	0QK	0.407	0.125	0.322	4.599	-6.108	3D PDB	Do
71	4abk	Transferase	POLY [ADP-RIBOSE] POLYMERASE 14	AR6	0.406	0.125	0.321	4.599	-6.446	3D PDB	Do
71	4z07	Kinase	cGMP-dependent protein kinase 1	PCG	0.459	0	0.321	5.702	-4.772	3D PDB	Do

Rank	PDB	Target Class	Target Name	Ligand Name	Ligand Similarity Score	Binding Similarity Score	LigTMap Score	Predicted Affinity (-log M)	PSOVina2 Docking Score (kcal/mol)	PSOVina2 Docking Pose	Selec bind simil anal
72	4nxv	Transferase	Mitochondrial dynamic protein MID51	GDP	0.424	0.077	0.32	4.599	-6.075	3D PDB	Do
72	1y8o	Kinase	[Pyruvate dehydrogenase [lipoamide]] kinase isozyme 3	ADP	0.43	0.063	0.32	5.702	-5.825	3D PDB	Do
73	3vfq	Transferase	Poly [ADP-ribose] polymerase 14	AR6	0.406	0.111	0.317	4.599	-6.898	3D PDB	Do
73	4bj9	Transferase	TANKYRASE-2	UHB	0.422	0.071	0.317	4.599	-5.907	3D PDB	Do
74	3nba	Tuberculosis	Phosphopantetheine adenylyltransferase	APC	0.422	0.063	0.314	4.685	-6.209	3D PDB	Do
75	4lps	Hpyroli	Hydrogenase/urease nickel incorporation protein HypB	GDP	0.424	0.056	0.313	5.28	-5.293	3D PDB	Do
76	4y62	Transferase	Histo-blood group ABO system transferase	48E	0.443	0	0.31	4.599	-5.164	3D PDB	Do
77	2l8r	Hydrolase	Uncharacterized protein C6orf130	APR	0.406	0.077	0.307	6.004	-7.029	3D PDB	Do
78	5htc	Kinase	Serine/threonine-protein kinase haspin	66M	0.433	0	0.303	5.702	-5.602	3D PDB	Do
79	3qxh	Hpyroli	Dethiobiotin synthetase	ADP	0.43	0	0.301	5.28	-5.523	3D PDB	Do
79	3llm	Hydrolase	ATP-dependent RNA helicase A	ADP	0.43	0	0.301	6.004	-4.374	3D PDB	Do
80	1h1h	Hydrolase	EOSINOPHIL CATIONIC PROTEIN	A2P	0.426	0	0.298	6.004	-4.727	3D PDB	Do
81	4q46	Influenza	Polymerase basic protein 2	GDP	0.424	0	0.297	7.773	-4.693	3D PDB	Do
82	3b2t	Transferase	Fibroblast growth factor receptor 2	M33	0.423	0	0.296	4.599	-5.829	3D PDB	Do
83	3lf0	Tuberculosis	Nitrogen regulatory protein P-II	ATP	0.422	0	0.295	4.685	-4.274	3D PDB	Do
83	4tk5	Transferase	Tankyrase-2	UHB	0.422	0	0.295	4.599	-5.88	3D PDB	Do

Rank	PDB	Target Class	Target Name	Ligand Name	Ligand Similarity Score	Binding Similarity Score	LigTMap Score	Predicted Affinity (-log M)	PSOVina2 Docking Score (kcal/mol)	PSOVina2 Docking Pose	Selec[illegible] bind[illegible] simil[illegible] anal[illegible]
84	1i5r	Beta_secretase	TYPE 1 17 BETA-HYDROXYSTEROID DEHYDROGENASE	HYC	0.403	0.037	0.293	5.476	-5.957	3D PDB	Do[illegible]
85	4fl1	Kinase	Tyrosine-protein kinase SYK	ANP	0.416	0	0.291	5.702	-5.001	3D PDB	Do[illegible]

Copyright © 2022 University of Macau

0[illegible]
